# Hospitalization of Infants and Children Aged 0–4 Years with Laboratory-Confirmed COVID-19 — COVID-NET, 14 States, March 2020–February 2022

**DOI:** 10.15585/mmwr.mm7111e2

**Published:** 2022-03-18

**Authors:** Kristin J. Marks, Michael Whitaker, Nickolas T. Agathis, Onika Anglin, Jennifer Milucky, Kadam Patel, Huong Pham, Pam Daily Kirley, Breanna Kawasaki, James Meek, Evan J. Anderson, Andy Weigel, Sue Kim, Ruth Lynfield, Susan L. Ropp, Nancy L. Spina, Nancy M. Bennett, Eli Shiltz, Melissa Sutton, H. Keipp Talbot, Andrea Price, Christopher A. Taylor, Fiona P. Havers, COVID-NET Surveillance Team, COVID-NET Surveillance Team, Jeremy Roland, Jordan Surgnier, Carol Lyons, Kyle Openo, Kenzie Teno, Alexander Kohrman, Erica Bye, Cory Cline, Alison Muse, Virginia Cafferky, Laurie Billing, Nasreen Abdullah, William Schaffner, Keegan McCaffrey

**Affiliations:** ^1^CDC COVID-19 Emergency Response Team; ^2^Epidemic Intelligence Service, CDC; ^3^General Dynamics Information Technology, Atlanta, Georgia; ^4^California Emerging Infections Program, Oakland, California; ^5^Colorado Department of Public Health and Environment; ^6^Connecticut Emerging Infections Program, Yale School of Public Health, New Haven, Connecticut; ^7^Emory University School of Medicine, Atlanta, Georgia; ^8^Georgia Emerging Infections Program, Georgia Department of Public Health; ^9^Atlanta Veterans Affairs Medical Center, Atlanta, Georgia; ^10^Iowa Department of Public Health; ^11^Michigan Department of Health and Human Services; ^12^Minnesota Department of Health; ^13^New Mexico Department of Health; ^14^New York State Department of Health; ^15^University of Rochester School of Medicine and Dentistry, Rochester, New York; ^16^Ohio Department of Health; ^17^Public Health Division, Oregon Health Authority; ^18^Vanderbilt University Medical Center, Nashville, Tennessee; ^19^Salt Lake County Health Department, Salt Lake City, Utah.; California Emerging Infections Program; Colorado Department of Public Health and Environment; Connecticut Emerging Infections Program, Yale School of Public Health; Georgia Emerging Infections Program, Georgia Department of Health, Division of Infectious Diseases, Emory University School of Medicine; Iowa Department of Health; Michigan Department of Health and Human Services; Minnesota Department of Health; New Mexico Department of Health; New York State Department of Health; University of Rochester School of Medicine and Dentistry; Ohio Department of Health; Public Health Division, Oregon Health Authority; Vanderbilt University Medical Center; Utah Department of Health

The B.1.1.529 (Omicron) variant of SARS-CoV-2, the virus that causes COVID-19, has been the predominant circulating variant in the United States since late December 2021.* Coinciding with increased Omicron circulation, COVID-19–associated hospitalization rates increased rapidly among infants and children aged 0–4 years, a group not yet eligible for vaccination (*1*). Coronavirus Disease 19–Associated Hospitalization Surveillance Network (COVID-NET)^†^ data were analyzed to describe COVID-19–associated hospitalizations among U.S. infants and children aged 0–4 years since March 2020. During the period of Omicron predominance (December 19, 2021–February 19, 2022), weekly COVID-19–associated hospitalization rates per 100,000 infants and children aged 0–4 years peaked at 14.5 (week ending January 8, 2022); this Omicron-predominant period peak was approximately five times that during the period of SARS-CoV-2 B.1.617.2 (Delta) predominance (June 27–December 18, 2021, which peaked the week ending September 11, 2021).^§^ During Omicron predominance, 63% of hospitalized infants and children had no underlying medical conditions; infants aged <6 months accounted for 44% of hospitalizations, although no differences were observed in indicators of severity by age. Strategies to prevent COVID-19 among infants and young children are important and include vaccination among currently eligible populations (*2*) such as pregnant women (*3*), family members, and caregivers of infants and young children (*4*).

COVID-NET conducts population-based surveillance for laboratory-confirmed COVID-19–associated hospitalizations in 99 counties across 14 U.S. states.^¶^ Among residents of a predefined surveillance catchment area, COVID-19–associated hospitalizations are defined as receipt of a positive SARS-CoV-2 real-time reverse transcription–polymerase chain reaction or rapid antigen detection test result during hospitalization or during the 14 days preceding admission. This analysis describes weekly hospitalization rates among infants and children aged 0–4 years during March 1, 2020–February 19, 2022, which includes the pre-Delta–, Delta- and Omicron-predominant periods; detailed clinical data were available through January 31, 2022. Unadjusted weekly COVID-19–associated hospitalization rates were calculated by dividing the total number of hospitalized patients by the population estimates within each age group for the counties included in the surveillance catchment area.** All rates are estimated per 100,000 infants and children aged 0–4 years. Rate ratios (RR) comparing Omicron- and Delta-predominant periods and 95% CIs were calculated. Three-week moving averages are presented for visualization purposes.

Trained surveillance staff conducted medical chart abstractions for all pediatric COVID-NET patients using a standardized case report form during March 2020 through November 2021. Because of the large surge in hospitalizations during December 2021 and January 2022, some sites examined clinical data on a representative sample of hospitalized infants and children.^††^ Data regarding primary reason for hospital admission,^§§^ symptoms at admission,^¶¶^ underlying medical conditions, and indicators of severe disease (i.e., hospital length of stay, intensive care unit [ICU] admission, need for respiratory support,*** and in-hospital death) were collected (*5*). Data on viral codetections (respiratory syncytial virus [RSV], influenza, rhinovirus/enterovirus, and other viruses)^†††^ were collected for infants and children who received additional testing. Monthly ICU admission rates were calculated. Proportions were compared between periods of pre-Delta predominance (March 1, 2020–June 26, 2021), Delta predominance (June 27–December 18, 2021), and Omicron predominance (December 19, 2021–January 31, 2022); a variant that accounted for >50% of sequenced isolates was considered predominant. For the period of Omicron predominance, proportions were compared by age (<6 months, 6–23 months, and 2–4 years). Wilcoxon rank-sum tests and chi-square tests were used to compare medians and proportions, respectively; p-values <0.05 were considered statistically significant. Percentages were weighted to account for the probability of selection for sampled cases and adjusted to account for nonresponse. Data were analyzed using SAS (version 9.4; SAS Institute). This activity was reviewed by CDC and was conducted consistent with applicable federal law and CDC policy.^§§§^

During March 1, 2020–February 19, 2022, weekly hospitalization rates (hospitalized patients per 100,000 infants and children aged 0–4 years) peaked during Omicron predominance, in the week ending January 8, 2022, at 14.5. This peak hospitalization rate during Omicron predominance was approximately five times the peak during Delta predominance (2.9) (week ending September 11, 2021) (RR = 5.0; 95% CI = 3.8–6.8). Hospitalization rates among infants aged <6 months were approximately six times as high during the peak week of Omicron predominance (68.1) as during Delta predominance (11.1) (RR = 6.1; 95% CI = 3.9–10.0); Omicron-predominant versus Delta-predominant hospitalization RRs were also elevated among infants and children aged 6–23 months (16.9 versus 3.3; RR = 5.1; 95% CI = 3.1–8.5) and 2–4 years (4.7 versus 1.4; RR = 3.5; 95% CI = 2.0–6.3) (Figure). Monthly ICU admission rates were approximately 3.5 times as high during the Omicron predominance peak in January 2022 (10.6) as during the Delta predominance peak in September 2021 (3.0). Hospitalization rates among infants and children aged 0–4 years decreased by the week ending February 19, 2022 (3.9).

**FIGURE:**
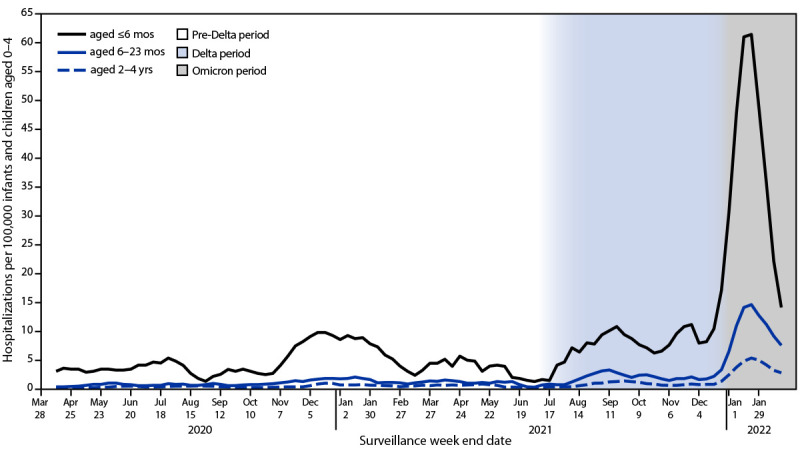
COVID-19–associated hospitalization rates* among infants and children aged 0–4 years, by age group (3-week moving average) — Coronavirus Disease 2019–Associated Hospitalization Surveillance Network, 14 states,^†^ March 2020–February 2022^§^ **Abbreviation**: COVID-NET = Coronavirus Disease 2019–Associated Hospitalization Surveillance Network. * Number of patients with laboratory-confirmed COVID-19–associated hospitalizations per 100,000 population; rates are subject to change as additional data are reported. ^†^ COVID-NET sites are in the following 14 states: California, Colorado, Connecticut, Georgia, Iowa, Maryland, Michigan, Minnesota, New Mexico, New York, Ohio, Oregon, Tennessee, and Utah. Starting the week ending December 4, 2021, Maryland data are removed from weekly rate calculations. ^§^ Periods of predominance are defined as follows: pre-Delta = March 1, 2020–June 26, 2021; Delta = June 27–December 18, 2021; Omicron = December 19, 2021–February 19, 2022.

Complete clinical data were available for 97% (2,562 of 2,637) of hospitalized infants and children aged 0–4 years, including 99% (1,200 of 1,209), 94% (790 of 841), and 97% (572 of 587) hospitalized during the pre-Delta–, Delta- and Omicron-predominant periods, respectively (Table 1). Although there was some variation across periods, most patients had COVID-19–related symptoms recorded at admission (87%) and COVID-19 as the primary reason for admission (85%). During Omicron predominance, 37% of hospitalized infants and children had one or more underlying medical condition.^¶¶¶^ Among 62% (1,582 of 2,562) of infants and children with testing for additional viral pathogens,**** the proportion hospitalized with RSV codetections was significantly higher during Delta predominance (20%) than during Omicron predominance (7%) (p<0.001). Compared with Delta predominance, hospital length of stay during Omicron predominance was shorter (2 versus 1.5 days, p = 0.002) and the proportion of hospitalized infants and children requiring ICU admission (27% versus 21%, p = 0.02) was lower.

**TABLE 1:** Demographic and clinical characteristics and outcomes among infants and children aged 0–4 years hospitalized with laboratory-confirmed COVID-19,* by variant predominance period — Coronavirus Disease 2019–Associated Hospitalization Surveillance Network, 14 states,^†^ March 1, 2020–January 31, 2022

Characteristic	Variant predominant period, no. (%) of hospitalizations				P-value^§^ (Omicron versus pre-Delta)	P-value^§^ (Omicron versus Delta)
	Total	Pre-Delta Mar 1, 2020–Jun 26, 2021	Delta Jun 27–Dec 18, 2021	Omicron Dec 19, 2021–Jan 31, 2022		
**Total no. of hospitalized infants and children**	**2,562^¶^**	**1,200^¶^**	**790^¶^**	**572^¶^**	**NA**	**NA**
**Age group, yrs, median (IQR)**	**0.6 (0.1–1.0)**	0.6 (0.1–1.1)	0.7 (0.1–1.1)	0.6 (0.1–1.0)	0.41	0.69
<6 months	**1,137 (44.3)**	547 (45.6)	338 (42.8)	252 (43.9)	0.46	0.76
6–23 months	**772 (30.4)**	345 (28.8)	247 (31.2)	180 (32.0)		
2–4 years	**653 (25.3)**	308 (25.6)	205 (26.0)	140 (24.1)		
**Sex**						
Male	**1,433 (56.1)**	651 (54.4)	443 (56.3)	339 (58.2)	0.18	0.54
Female	**1,129 (43.9)**	549 (45.6)	347 (43.7)	233 (41.8)		
Race/Ethnicity**						
Hispanic	**710 (28.8)**	397 (32.9)	184 (24.2)	129 (27.5)	0.001	0.40
Black, non-Hispanic	**719 (26.7)**	347 (28.8)	219 (27.5)	153 (23.1)		
White, non-Hispanic	**767 (29.9)**	283 (23.5)	278 (34.6)	206 (34.1)		
Asian or other Pacific Islander, non-Hispanic	**154 (6.0)**	76 (6.5)	45 (5.6)	33 (5.7)		
Persons of all other races^††^	**65 (2.8)**	32 (2.7)	16 (2.2)	17 (3.4)		
Unknown race/ethnicity	**147 (5.8)**	65 (5.5)	48 (5.9)	34 (6.1)		
**Primary reason for admission^§§^**						
Likely COVID-19–related	**2,068 (84.7)**	874 (80.5)	709 (90.0)	485 (84.8)	0.06	0.009
**COVID-19–related symptoms at admission^¶¶^**						
Yes	**2,217 (86.6)**	1,000 (83.6)	715 (90.8)	502 (86.9)	0.13	0.04
**Underlying medical conditions**						
One or more underlying medical condition***	**923 (35.8)**	412 (34.6)	291 (36.8)	220 (36.6)	0.45	0.95
Prematurity^†††^	**294 (15.5)**	120 (13.3)	100 (17.0)	74 (17.1)	0.10	0.95
Neurologic disorders	**270 (10.3)**	134 (11.0)	76 (9.5)	60 (10.0)	0.56	0.78
Chronic lung disease, including asthma	**202 (7.7)**	93 (7.9)	74 (9.4)	35 (5.8)	0.13	0.02
Congenital heart disease	**152 (6.3)**	62 (5.2)	41 (5.2)	49 (8.6)	0.01	0.02
Immunocompromised condition	**81 (3.2)**	40 (3.3)	23 (2.9)	18 (3.2)	0.92	0.83
Chronic lung disease of prematurity/BPD	**64 (2.5)**	27 (2.3)	19 (2.5)	18 (2.6)	0.67	0.86
Abnormality of airway	**63 (2.3)**	40 (3.4)	12 (1.5)	11 (1.4)	0.01	0.91
Chronic metabolic disease	**61 (2.3)**	31 (2.5)	15 (1.8)	15 (2.5)	0.95	0.39
**Viral codetection^§§§^**						
RSV	**154 (9.5)**	9 (1.6)	115 (19.7)	30 (7.3)	<0.001	<0.001
Influenza	**11 (0.7)**	1 (0.2)	3 (0.5)	7 (1.3)	0.02	0.16
Rhinovirus/Enterovirus	**203 (17.0)**	66 (15.1)	103 (25.8)	34 (10.7)	0.10	<0.001
Other viral infection	**103 (8.8)**	30 (6.5)	45 (11.2)	28 (9.0)	0.23	0.35
**Hospitalization outcome^¶¶¶^**						
Length of hospital stay, days, median (IQR)	**1.5 (1–3.0)**	1.5 (1–3.5)	2 (1–3.5)	1.5 (0.5–2.5)	0.001	0.002
ICU admission	**624 (23.9)**	290 (24.0)	210 (26.7)	124 (21.0)	0.19	0.02
BiPAP/CPAP	**172 (6.5)**	69 (5.9)	72 (9.1)	31 (5.1)	0.53	0.008
High flow nasal cannula	**341(13.3)**	98 (8.3)	159 (20.4)	84 (13.4)	0.002	0.002
Invasive mechanical ventilation	**146 (5.7)**	77 (6.4)	40 (5.2)	29 (5.2)	0.39	0.96
In-hospital death	**16 (0.6)**	10 (0.8)	4 (0.5)	2 (0.5)	0.51	0.99

**Abbreviations**: BiPAP/CPAP = bilevel positive airway pressure/continuous positive airway pressure; BPD = bronchopulmonary dysplasia; COVID-NET = Coronavirus Disease 2019–Associated Hospitalization Surveillance Network; ICU = intensive care unit; NA = not applicable; RSV = respiratory syncytial virus.

* Data are from a weighted sample of hospitalized infants and children with completed medical record abstractions. Sample sizes presented are unweighted with weighted percentages.

^†^ Includes persons admitted to a hospital with an admission date during March 1, 2020–January 31, 2022. Maryland contributed data through November 26, 2021. Counties included in COVID-NET surveillance: California (Alameda, Contra Costa, and San Francisco counties); Colorado (Adams, Arapahoe, Denver, Douglas, and Jefferson counties); Connecticut (Middlesex and New Haven counties); Georgia (Clayton, Cobb, DeKalb, Douglas, Fulton, Gwinnett, Newton, and Rockdale counties); Iowa (one county represented); Maryland (Allegany, Anne Arundel, Baltimore, Baltimore City, Calvert, Caroline, Carroll, Cecil, Charles, Dorchester, Frederick, Garrett, Harford, Howard, Kent, Montgomery, Prince George’s, Queen Anne’s, St. Mary’s, Somerset, Talbot, Washington, Wicomico, and Worcester counties); Michigan (Clinton, Eaton, Genesee, Ingham, and Washtenaw counties); Minnesota (Anoka, Carver, Dakota, Hennepin, Ramsey, Scott, and Washington counties); New Mexico (Bernalillo, Chaves, Doña Ana, Grant, Luna, San Juan, and Santa Fe counties); New York (Albany, Columbia, Genesee, Greene, Livingston, Monroe, Montgomery, Ontario, Orleans, Rensselaer, Saratoga, Schenectady, Schoharie, Wayne, and Yates counties); Ohio (Delaware, Fairfield, Franklin, Hocking, Licking, Madison, Morrow, Perry, Pickaway and Union counties); Oregon (Clackamas, Multnomah, and Washington counties); Tennessee (Cheatham, Davidson, Dickson, Robertson, Rutherford, Sumner, Williamson, and Wilson counties); and Utah (Salt Lake county).

^§^ Proportions between the Omicron and Delta and Omicron and pre-Delta predominance periods were compared using chi-square tests, and medians were compared using Wilcoxon rank-sum tests; p-values <0.05 were considered statistically significant.

^¶^ Data are missing for <6% of observations for all variables, except for viral codetections.

** If ethnicity was unknown, non-Hispanic ethnicity was assumed.

^††^ Includes non-Hispanic persons reported as other or multiple races.

^§§^ Primary reason for admission was collected beginning June 1, 2020; hospitalizations before June 1, 2020, are excluded. Among sampled patients, COVID-NET collects data on the primary reason for admission to differentiate hospitalizations of patients with laboratory-confirmed SARS-CoV-2 infection who are likely admitted primarily for COVID-19 illness versus other reasons. During chart review, if the surveillance officer finds that the chief complaint or history of present illness mentions fever/respiratory illness, COVID-19–like illness, or a suspicion for COVID-19, then the case is categorized as COVID-19–related illness as the primary reason for admission. Reasons for admission that are likely primarily not COVID-19–related include categories such as inpatient surgery or trauma. Infants diagnosed with COVID-19 during their birth hospitalization were not categorized as likely COVID-19–related unless they exhibited COVID-19–related symptoms.

^¶¶^ COVID-19–related symptoms included respiratory symptoms (congested/runny nose, cough, hemoptysis/bloody sputum, shortness of breath/respiratory distress, sore throat, upper respiratory infection, influenza-like illness, and wheezing) and non-respiratory symptoms (abdominal pain, altered mental status/confusion, anosmia/decreased smell, chest pain, conjunctivitis, diarrhea, dysgeusia/decreased taste, fatigue, fever/chills, headache, muscle aches/myalgias, nausea/vomiting, rash, and seizures, and among those aged <2 years: apnea, cyanosis, decreased vocalization/stridor, dehydration, hypothermia, inability to eat/poor feeding, and lethargy). Symptoms are abstracted from the medical chart and might be incomplete.

*** Defined as one or more of the following: chronic lung disease, chronic metabolic disease, blood disorder/hemoglobinopathy, cardiovascular disease, neurologic disorder, immunocompromised condition, renal disease, gastrointestinal/liver disease, rheumatologic/autoimmune/inflammatory condition, obesity, feeding tube dependency, or wheelchair dependency.

^†††^ Prematurity as an underlying medical condition is only reported for infants and children aged <2 years.

^§§§^ Results reported among infants and children who had testing performed (as opposed to all hospitalized infants and children). Because of testing practices, denominators differed among the viral respiratory pathogens: 1,582 infants and children were tested for RSV, 1,644 for influenza (influenza A, influenza B, flu [not subtyped]), 1,109 for rhino/enterovirus, and 1,120 for other viruses (adenovirus, parainfluenza 1, parainfluenza 2, parainfluenza 3, parainfluenza 4, human metapneumovirus).

^¶¶¶^ Hospitalization outcomes are not mutually exclusive; patients could be included in more than one category.

During Omicron predominance, 44% of hospitalized infants and children aged 0–4 years were infants aged <6 months, similar to proportions during the Delta- (43%) and pre-Delta–predominant (46%) periods. Among 252 hospitalized infants aged <6 months, 146 (58%) were aged <2 months, 30 (21%) of whom received a diagnosis of COVID-19 during their birth hospitalization. A smaller proportion of infants and children aged <6 months was hospitalized with COVID-19–related symptoms at admission (82%) than the proportion aged 6–23 months (92%) or 2–4 years (89%) (Table 2), although no difference was observed when birth hospitalizations (91% of which were asymptomatic infections) were excluded. Approximately one half (51%) of hospitalized infants aged <6 months were febrile at the time of admission, including 44% of those aged <2 months and 61% of those aged 2–5 months. A higher proportion of infants aged <6 months (13%) were hospitalized with RSV codetections than were older infants and children (6–23 months = 4%; 2–4 years = 2%). Length of stay, ICU admission, and need for respiratory support did not significantly differ by age group.

**TABLE 2:** Clinical characteristics and outcomes among infants and children aged 0–4 years hospitalized with laboratory-confirmed COVID-19 (N = 572),* by age group, during Omicron predominance — COVID-NET, 14 states,^†^ December 19, 2021–January 31, 2022

Characteristic	No. (%) of hospitalizations, by age group				P-value^§^
	Total	<6 mos	6–23 mos	2–4 yrs	
**Total no. of hospitalized infants and children**	**572 (100)^¶^**	**252 (44)^¶^**	**180 (32)^¶^**	**140 (24)^¶^**	**NA**
Primary reason for admission**					
Likely COVID-19–related	**485 (84.8)**	210 (83.3)	159 (89.2)	116 (81.8)	0.23
**COVID-19–related symptoms at admission^††^**					
Yes	**502 (86.9)**	211 (82.0)^§§^	163 (91.9)	128 (89.2)	0.04
**Symptoms at admission**					
Fever/chills	**340 (60.3)**	128 (51.0)	123 (70.8)	89 (63.2)	0.001
Cough	**317 (55.6)**	119 (45.6)	120 (70.8)	78 (53.7)	<0.001
Congested/Runny nose	**290 (52.1)**	135 (51.3)	98 (61.1)	57 (41.6)	0.01
Shortness of breath/Respiratory distress	**201 (34.7)**	85 (31.0)	74 (43.8)	42 (29.3)	0.02
Inability to eat/Poor feeding	**139 (29.2)**	75 (26.6)	64 (32.6)	—^¶¶^	0.21
Nausea/Vomiting	**148 (26.6)**	40 (18.1)	59 (31.8)	49 (35.4)	0.003
Fatigue	**83 (13.4)**	21 (6.6)	25 (13.7)	37 (25.2)	<0.001
Decreased vocalization/Stridor	**49 (11.6)**	15 (5.8)	34 (19.7)	—^¶¶^	<0.001
Seizures	**27 (3.9)**	4 (1.5)	9 (5.0)	14 (6.9)	0.02
**Underlying medical condition**					
One or more underlying medical condition***	**220 (36.6)**	66 (26.3)	80 (40.3)	74 (50.4)	<0.001
Prematurity	**74 (17.1)**	39 (16.7)	35 (17.7)	—^¶¶^	0.83
Neurologic disorders	**60 (10.0)**	10 (3.6)	17 (8.9)	33 (23.0)	<0.001
Congenital heart disease	**49 (8.6)**	18 (7.1)	19 (9.2)	12 (10.5)	0.62
Chronic lung disease, including asthma	**35 (5.8)**	5 (2.5)	12 (5.3)	18 (12.6)	<0.001
Immunocompromised condition	**18 (3.2)**	1 (0.5)	5 (1.9)	12 (9.7)	<0.001
Chronic lung disease of prematurity/BPD	**18 (2.6)**	4 (1.8)	7 (2.6)	7 (4.3)	0.32
Chronic metabolic disease	**15 (2.5)**	2 (0.7)	5 (2.8)	8 (5.3)	0.02
Abnormality of airway	**11 (1.4)**	4 (1.2)	5 (1.8)	2 (1.3)	0.85
**Viral codetection^†††^**					
RSV	**30 (7.3)**	22 (12.7)	6 (4.3)	2 (2.0)	0.003
Influenza	**7 (1.3)**	4 (1.3)	1 (0.8)	2 (2.1)	0.62
Rhinovirus/Enterovirus	**34 (10.7)**	13 (10.6)	10 (8.4)	11 (13.5)	0.59
Other viral infections	**28 (9.0)**	4 (3.2)	14 (13.4)	10 (12.2)	0.03
**Hospitalization outcome^§§§^**					
Length of hospital stay, days, median (IQR)	**1.5 (0.5–2.5)**	1.5 (1–2.5)	1.5 (0.5–3)	1.5 (0.5–3)	0.70
ICU admission	**124 (21.0)**	57 (21.6)	39 (21.9)	28 (18.9)	0.81
BiPAP/CPAP	**31 (5.1)**	12 (4.5)	12 (6.1)	7 (4.8)	0.76
High flow nasal cannula	**84 (13.4)**	43 (14.1)	28 (16.1)	13 (8.7)	0.20
Invasive mechanical ventilation	**29 (5.2)**	10 (4.6)	11 (5.9)	8 (5.6)	0.84
In-hospital death	**2 (0.5)**	2 (1.1)	0 (—)	0 (—)	0.70

**Abbreviations**: BiPAP/CPAP = bilevel positive airway pressure/continuous positive airway pressure; BPD = bronchopulmonary dysplasia; COVID-NET = Coronavirus Disease 2019–Associated Hospitalization Surveillance Network; ICU = intensive care unit; NA = not applicable; RSV = Respiratory syncytial virus.

* Data are from a weighted sample of hospitalized infants and children with completed medical record abstractions. Sample sizes presented are unweighted with weighted percentages.

^†^ Includes persons admitted to a hospital with an admission date during December 19, 2021–January 31, 2022. Counties included in COVID-NET surveillance during this period: California (Alameda, Contra Costa, and San Francisco counties); Colorado (Adams, Arapahoe, Denver, Douglas, and Jefferson counties); Connecticut (Middlesex and New Haven counties); Georgia (Clayton, Cobb, DeKalb, Douglas, Fulton, Gwinnett, Newton, and Rockdale counties); Iowa (one county represented); Michigan (Clinton, Eaton, Genesee, Ingham, and Washtenaw counties); Minnesota (Anoka, Carver, Dakota, Hennepin, Ramsey, Scott, and Washington counties); New Mexico (Bernalillo, Chaves, Doña Ana, Grant, Luna, San Juan, and Santa Fe counties); New York (Albany, Columbia, Genesee, Greene, Livingston, Monroe, Montgomery, Ontario, Orleans, Rensselaer, Saratoga, Schenectady, Schoharie, Wayne, and Yates counties); Ohio (Delaware, Fairfield, Franklin, Hocking, Licking, Madison, Morrow, Perry, Pickaway and Union counties); Oregon (Clackamas, Multnomah, and Washington counties); Tennessee (Cheatham, Davidson, Dickson, Robertson, Rutherford, Sumner, Williamson, and Wilson counties); and Utah (Salt Lake county).

^§^ Proportions of infants and children aged <6 months, 6–23 months, and 2–4 years were compared using chi-square tests, and medians were compared using the Wilcoxon rank-sum test; p-values <0.05 were considered statistically significant.

^¶^ Data are missing for <6% of observations for all variables, except for viral codetections.

** Among sampled patients, COVID-NET collects data on the primary reason for admission to differentiate hospitalizations of patients with laboratory-confirmed SARS-CoV-2 infection who are likely admitted primarily for COVID-19 illness versus other reasons. During chart review, if the surveillance officer found that the chief complaint or history of present illness mentions fever/respiratory illness, COVID-19–like illness, or a suspicion for COVID-19, then the case was categorized as COVID-19–related illness as the primary reason for admission. Reasons for admission that are likely primarily not COVID-19–related include categories such as inpatient surgery or trauma. Infants with COVID-19 diagnosed during their birth hospitalization were not categorized as likely COVID-19–related unless they exhibited COVID-19–related symptoms.

^††^ COVID-19–related symptoms included respiratory symptoms (congested/runny nose, cough, hemoptysis/bloody sputum, shortness of breath/respiratory distress, sore throat, upper respiratory infection, influenza-like illness, and wheezing) and non-respiratory symptoms (abdominal pain, altered mental status/confusion, anosmia/decreased smell, chest pain, conjunctivitis, diarrhea, dysgeusia/decreased taste, fatigue, fever/chills, headache, muscle aches/myalgias, nausea/vomiting, rash, and seizures, and among those aged <2 years: apnea, cyanosis, decreased vocalization/stridor, dehydration, hypothermia, inability to eat/poor feeding, and lethargy). Symptoms are abstracted from the medical chart and might be incomplete.

^§§^ Among the 250 hospitalizations among infants aged <6 months with complete data on birth hospitalization, 14% (31 of 250) were birth hospitalizations. Of these birth hospitalizations, 91% (28 of 31) had no symptoms recorded. If birth hospitalizations are excluded, 94% (208 of 219) infants aged <6 months had symptoms recorded.

^¶¶^ Cyanosis, decreased vocalization/stridor, inability to eat/poor feeding, and lethargy are symptoms that are only recorded for infants and children aged <2 years. Prematurity is an underlying medical condition only reported for infants and children aged <2 years.

*** Defined as one or more of the following: chronic lung disease, chronic metabolic disease, blood disorder/hemoglobinopathy, cardiovascular disease, neurologic disorder, immunocompromised condition, renal disease, gastrointestinal/liver disease, rheumatologic/autoimmune/inflammatory condition, obesity, feeding tube dependency, or wheelchair dependency.

^†††^ Results reported among infants and children who had testing performed (as opposed to all hospitalized infants and children). Because of differing testing practices, denominators differed among the viral respiratory pathogens: 424 infants and children were tested for RSV, 440 for influenza (influenza A, influenza B, flu [not subtyped]), 260 for rhino/enterovirus, and 261 for other viruses (adenovirus, parainfluenza 1, parainfluenza 2, parainfluenza 3, parainfluenza 4, and human metapneumovirus).

^§§§^ Hospitalization outcomes are not mutually exclusive; patients could be included in more than one category.

## Discussion

Weekly COVID-19–associated hospitalization rates among U.S. infants and children aged 0–4 years have declined since the peak of January 8, 2022; however, peak rates during Omicron predominance were approximately five times those of the peak during Delta predominance. Similarly, ICU admission rates during Omicron predominance peaked at approximately 3.5 times the peak rate during Delta predominance. The proportion of hospitalized infants and children with severe illness during all variant periods of predominance, coupled with the potential for longer-term sequelae including multisystem inflammatory syndrome (*6**,**7*), highlight the importance of preventing COVID-19 among infants and children aged 0–4 years. Strategies to prevent COVID-19 among infants and young children are important and include vaccination of currently eligible populations (*2*) such as pregnant women (*3*), family members, and caregivers of infants and young children (*4*).

The proportion of patients with codetections of RSV was higher during Delta predominance than Omicron predominance. RSV circulation was low during the first year of the pandemic (pre-Delta predominance). The pattern of RSV codetections during 2021–2022 correlated with trends in RSV circulation observed in other surveillance systems: RSV circulation increased during the summer and fall of 2021 (Delta predominance) and declined during Omicron predominance (*8*).^††††^ These limited data suggest that the surge in hospitalizations during Omicron predominance was not driven by coinfections. The highest proportion of hospitalized infants and children requiring ICU admission occurred during Delta predominance, and the lowest occurred during Omicron predominance. Although the proportion of hospitalized infants and children admitted to an ICU was higher during Delta predominance, the rate of pediatric ICU admissions during Omicron predominance was approximately 3.5 times as high as that during Delta predominance, driven by the overall higher disease incidence.

Throughout the pandemic, infants aged <6 months have been hospitalized with laboratory-confirmed COVID-19 at higher rates than have infants and children aged 6 months–4 years. Infants aged <6 months were hospitalized with RSV codetections in higher proportions but required ICU admission and respiratory support in similar proportions to other age groups. Future studies are needed to understand the possible long-term consequences of COVID-19 infection among infants. Although infants aged <6 months are not currently eligible for vaccination, evidence suggests that this age group can receive protection through passive transplacental transfer of maternal antibodies acquired through vaccination (*9*). CDC recommends that women who are pregnant, breastfeeding, trying to become pregnant, or might become pregnant get vaccinated and stay up to date with COVID-19 vaccination.

The findings in this report are subject to at least four limitations. First, COVID-19–associated hospitalizations and viral coinfections might have been missed because of testing practice differences and test availability; this analysis could not account for changes in viral testing practices over time. Second, periods of variant predominance are not exclusive to a given variant; other variants might be circulating while one predominates. Third, it was not possible to account for seasonality or changes in public health policies and treatment practices over time; for example, the proportion of ICU admissions might reflect changing hospital capacity during the period of variant predominance rather than disease severity. Finally, the COVID-NET catchment areas include approximately 10% of the U.S. population; thus, these findings might not be nationally generalizable.

Coinciding with Omicron predominance, COVID-19–associated hospitalization rates among infants and children aged 0–4 years reached the current highest level of the pandemic during early January 2022. All persons who are eligible for vaccination (*2*), including pregnant women (*3*), should receive and stay up to date with COVID-19 vaccination to reduce the risk for severe disease for themselves and others with whom they come into contact (*10*), including infants and children aged 0–4 years who are currently not eligible for vaccination (*4*).

**Summary**

**What is already known about this topic?**

COVID-19 can cause severe illness in infants and children, including those aged 0–4 years who are not yet eligible for COVID-19 vaccination.

**What is added by this report?**

During Omicron variant predominance beginning in late December 2021, U.S. infants and children aged 0–4 years were hospitalized at approximately five times the rate of the previous peak during Delta variant predominance. Infants aged <6 months had the highest rates of hospitalization, but indicators of severity (e.g., respiratory support) did not differ by age group.

**What are the implications for public health practice?**

Important strategies to prevent COVID-19 among infants and young children include vaccination of currently eligible populations such as pregnant women, family members, and caregivers of infants and young children.

## Footnotes

* Delta became the predominant variant (>50% of sequenced isolates) circulating in the United States during the week ending July 3, 2021 (https://covid.cdc.gov/covid-data-tracker/#variant-proportions). By the week ending December 18, 2021, Omicron accounted for 38% of circulating variants; Omicron became the predominant variant during the week ending December 25 at 74%.

†
https://www.cdc.gov/coronavirus/2019-ncov/covid-data/covid-net/purpose-methods.html

§ COVID-NET hospitalization data are preliminary and subject to change as more data become available. Specifically, case counts and rates for recent hospital admissions are subject to reporting lag, and some data might be incomplete or unavailable for children with prolonged hospitalizations who might still be hospitalized at the time of publication.

¶ California, Colorado, Connecticut, Georgia, Iowa, Maryland, Michigan, Minnesota, New Mexico, New York, Ohio, Oregon, Tennessee, and Utah.

** Rates are calculated using the National Center for Health Statistics’ vintage 2020 bridged race postcensal population estimates for the counties included in surveillance (https://www.cdc.gov/nchs/nvss/bridged_race.htm).

†† During December 2021–January 2022, sites sampled pediatric patients at 12%–100%. Random numbers (1–100) are automatically generated and assigned to each patient on entry into the surveillance database to produce random samples of hospitalized patients for medical record abstraction. Percentages are weighted to account for the probability of selection for sampled patients.

§§ Among sampled cases, COVID-NET collects data on the primary reason for admission to differentiate hospitalizations of patients with laboratory-confirmed SARS-CoV-2 infection who are likely admitted primarily for COVID-19 illness versus other reasons, including inpatient surgery or trauma. Infants with diagnosed COVID-19 during their birth hospitalization were not categorized as likely COVID-19–related unless they exhibited COVID-19–related signs or symptoms.

¶¶ COVID-19–related symptoms included respiratory symptoms (congestion/runny nose, cough, hemoptysis/bloody sputum, shortness of breath/respiratory distress, sore throat, upper respiratory infection, influenza-like illness, and wheezing) and nonrespiratory symptoms (abdominal pain, altered mental status/confusion, anosmia/decreased smell, chest pain, conjunctivitis, diarrhea, dysgeusia/decreased taste, fatigue, fever/chills, headache, muscle aches/myalgias, nausea/vomiting, rash, and seizures, and among infants and children aged <2 years: apnea, cyanosis, decreased vocalization/stridor, dehydration, hypothermia, inability to eat/poor feeding, and lethargy). Symptoms were abstracted from medical charts and might be incomplete.

*** ICU admission and respiratory support are not mutually exclusive categories, and patients could have received both.

††† Testing is clinician-driven and proportions with codetections include infants and children who received a test in the denominator (as opposed to all hospitalized infants and children). Influenza includes influenza A, influenza B, and flu (not subtyped). Other viruses include adenovirus, parainfluenza 1, parainfluenza 2, parainfluenza 3, parainfluenza 4, and human metapneumovirus.

§§§ 45 C.F.R. part 46.102(l)(2), 21 C.F.R. part 56; 42 U.S.C. Sect. 241(d); 5 U.S.C. Sect. 552a; 44 U.S.C. Sect. 3501 et seq.

¶¶¶ Defined as one or more of the following: chronic lung disease, chronic metabolic disease, blood disorder/hemoglobinopathy, cardiovascular disease, neurologic disorder, immunocompromised condition, renal disease, gastrointestinal/liver disease, rheumatologic/autoimmune/inflammatory condition, obesity, feeding tube dependency, or wheelchair dependency.

**** Forty-eight percent (581 of 1,200) of infants and children received RSV testing during pre-Delta predominance (March 1, 2020–June 26, 2021), 73% (577 of 790) during Delta predominance (June 27–December 18, 2021), and 74% (424 of 572) during Omicron predominance (December 19, 2021–January 31, 2022).

††††
https://www.cdc.gov/surveillance/nrevss/rsv/natl-trend.html
